# Supplementary material for: Complexome profiling of the Chlamydomonas psb28 mutant reveals TEF5 as an early PSII assembly factor
Source: Plant Cell. 2025 Mar 17;37(6):koaf055. doi: 10.1093/plcell/koaf055 (PMC12164590; doi:10.1093/plcell/koaf055)
Supplement: koaf055_Supplementary_Data [file koaf055_supplementary_data.zip › psb28_interactive.zip!/plots/intensity/qualityPlots/peptides/SPP1A_Cre10.g444550.t1.2.html]

### Cre10.g444550.t1.2

Trivial name: SPP1A  
SeqCoverage [median]: 9.20  
Mass: 80.78 kDa  
  
 

| Sequence | MissedCleavage | Mass [Da] | wtIntensity | muIntensity | Score |
| --- | --- | --- | --- | --- | --- |
| FAELLADNR | 0 | 1047.53 | 0.00 | 1004.05 | 79.91 |
| HAAGIPAEEPVTVVELGK | 0 | 1815.97 | 762.94 | 3320.22 | 71.70 |
| LVDALGGVNR | 0 | 1012.57 | 261.34 | 1938.70 | 73.26 |
| QVSTALGK | 0 | 802.45 | 0.00 | 365.19 | 66.07 |
| TSGAIVGK | 0 | 731.42 | 0.00 | 360.74 | 91.91 |
| YSSVSPTAFPTLGTAGGK | 0 | 1739.87 | 0.00 | 475.20 | 38.37 |
|  |  |  | 1024.3 | 7464.1 |  |
